# Fracture luxation centrale bilatérale des deux hanches

**DOI:** 10.11604/pamj.2015.22.384.8610

**Published:** 2015-12-29

**Authors:** Mohamed Amine Karabila, Mustapha Mahfoud

**Affiliations:** 1 Service de Chirurgie Orthopédique et de Traumatologie, CHU Ibn Sina, Rabat, Maroc

**Keywords:** Fracture, cotyle, bilatérale, Fracture, acetabulum, bilateral

## Image en medicine

La fracture luxation centrale du cotyle est une lésion rare et nécessite un traumatisme violent, l'association de deux lésions similaires est encore plus rare, au mécanisme souvent imprécis et le choc doit être très violent, et donc elles se présentent dans le cadre de polytraumatisme, d'où l'intérêt de les rechercher systématiquement. Nous rapportons l'image d'un patient de 26 ans victime d'une chute d’échafaudage, présentant sur le plan locomoteur un traumatisme fermé des deux hanches. Le bilan radiographique a montré une fracture de la paroi postérieure des deux cotyles associée à une luxation postérieure des 2 têtes fémorales (A, B). Le patient a bénéficié en urgence d'une réduction orthopédique par traction des deux membres puis à j+3 d'une ostéosynthèse des deux cotyles en un seul temps par 2 voies de kocher langenbeck (C). À cinq mois, la récupération fonctionnelle des hanches est presque totale surtout à gauche. Le pronostic fonctionnel de ce type de lésion dépend de la précocité de la prise en charge.

**Figure 1:**
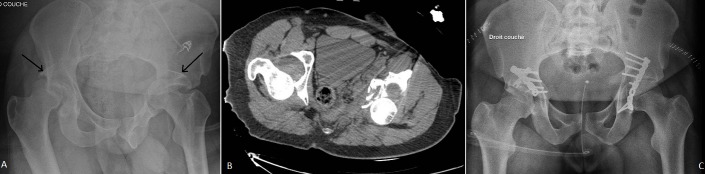
A) fracture luxation postérieure des 2 hanches; B) scanner du bassin montrant la luxation des 2 tètes associée à une fracture des 2 parois postérieures; C) ostéosynthèse des 2 hanches

